# Identification of crystallin modifications in the human lens cortex and nucleus using laser capture microdissection and CyDye labeling

**Published:** 2010-03-23

**Authors:** C.O. Asomugha, R. Gupta, O.P. Srivastava

**Affiliations:** Department of Vision Sciences, University of Alabama at Birmingham, Birmingham, AL

## Abstract

**Purpose:**

With aging, lens crystallins undergo post-translational modifications (PTMs) and these modifications are believed to play a major role in age-related cataract development. The purpose of the present study was to determine the protein profiles of crystallins and their PTMs in the cortical and nuclear regions within an aging human lens to gain a better understanding about changes in crystallins as fiber cells migrate from cortical to nuclear region.

**Methods:**

Laser capture microdissection (LCM) was used to select and capture cells from cortical and nuclear regions of 12 μm, optimum cutting temperature (OCT) compound-embedded frozen lens sections from a 69-year-old human lens. Proteins were extracted and then analyzed by 2-D difference gel electrophoresis (2-D DIGE) with sulfonated indocyanine dye (CyDye) labeling. Crystallin identities and their PTMs were then determined by Matrix-Assisted Laser Desorption/Ionization Time-of-Flight (MALDI-TOF) and Electrospray Ionization Quadripole Linear Ion-Trap Liquid Chromatography (ESI-QTRAP LC-MS/MS) mass spectrometry.

**Results:**

Crystallin fragments (M_r_ <20 kDa) were present in both cortical and nuclear regions, while high molecular weight (HMW) aggregates (M_r_ > 35 kDa) were mostly localized in the nuclear region. HMW complexes contained a relatively large number of truncated and modified β-crystallins, compared to α- and γ-crystallins, and two lens-specific intermediate filaments, CP49 (phakinin) and filensin. Modified α-crystallins were in low abundance in the nuclear region compared to the cortical region. Several PTMs, including deamidation, oxidation, phosphorylation, ethylation, methylation, acetylation, and carbamylation, were identified in virtually all crystallins and CP49. The data provide the first report of human lens crystallin profiling by a combination of LCM, 2D-DIGE, and mass spectrometric analysis.

**Conclusions:**

The results suggested that as the fiber cells migrate from cortical region to the nuclear region, the crystallin degradation begins in the cortical region and continues in the nuclear region. However, a greater number of the HMW complexes exist mainly in the nuclear region.

## Introduction

Crystallins (α, β, and γ) are structural proteins whose ability to form soluble oligomers in high concentrations, along with their specific short-range interactions, increase the refractive power of the lens and maintain lens transparency [1,2]. α-Crystallin is a large heterogenous oligomer, present in both epithelial and fiber cells, and composed of two primary gene products, αA- and αB-crystallin. In the β/γ-crystallin superfamily, β-crystallin has seven primary gene products – four acidic (βA1-, βA2-, βA3-, and βA4-crystallin) and three basic (βB1-, βB2-, and βB3-crystallin) – while there are seven γ-crystallins (γA- to γF- and γS-crystallin), with only γA-, γB-, γC-, γD-, and γS-crystallin present in humans [3-5]. As a member of a larger small heat shock protein (sHSP) superfamily, α-crystallin functions as a molecular chaperone [6] to prevent non-specific aggregation of improperly folded or denatured proteins [7,8], and is therefore believed to play a critical role in maintaining lens transparency.

Post-translational modifications (PTMs) that occur with aging are thought to be one of the causative factors in human cataract development because of their effects on crystallin structure and interactions. Recent genetic studies clearly demonstrated that the association of human inherited autosomal dominant, congenital zonular or nuclear sutural cataracts with misfolded proteins or prematurely terminated crystallins was the result of truncation at the translational level [9-11]. Several PTMs have already been identified in human lenses, including deamidation [12,13], oxidation of Trp, Met, and His residues [14,15], disulfide bonding [16], glycation [17], transglutaminase-mediated cross-linking [18], methylation [19], phosphorylation, and truncation of crystallins, and have been shown to alter crystallin stability, solubility, and function [19-22]. However, deamidation has been identified as the most commonly occurring modification [12,13,23-25].

Cataractous lenses have previously shown selective insolubilization of βA3/A1- and βB1-crystallin fragments and relatively greater truncation, deamidation of Asn residues, and oxidation of Trp [26,27]. This was consistent with earlier studies which showed that a major fraction of water soluble (WS) protein in adult human lens is composed of modified crystallin species, specifically truncated βB1- and βA3/A1-crystallin, and that almost all human crystallins are subject to deamidation [24,28]. Studies have also concluded that the major modifications distinguishing WS- and water insoluble (WI)-crystallins were increased, which included disulfide bonding, oxidation of Met, deamidation of Gln and Asn, truncation of both NH_2_- and COOH- termini of αA- and αB-crystallin, and backbone cleavage [29,30]. All of the above-mentioned studies used homogenized lens extracts. However, since homogenization of lenses in buffered solutions often solubilizes only part of the lens proteins [29], such studies that require whole tissue homogenates may not accurately represent molecular events taking place in the cortical and nuclear regions. Further, how the distribution of modified crystallin species changes during migration of fiber cells from the cortical to the nuclear region within an aging human lens has not been investigated. Consequences of the above mentioned PTMs on crystallin structure and function have been investigated extensively, but mainly with in vitro studies. For example, results have shown various modifications including deamidation at specific sites altered structural and oligomeric properties of βB1-, βB2- and other crystallins [22,30,31], as well as the chaperone activity of both αA- and αB-crystallin [20,21]. Our recent studies have shown that deletion of the COOH-terminal extension, but not the NH_2_-terminal domain, led to the insolubilization of αA-crystallin [32].

As an alternative approach to above in vitro studies, we used laser capture microdissection (LCM) and two-dimensional difference gel electrophoresis (2D-DIGE), which allow comparative expression following isolation of specific tissue/cells from the same tissue or identical regions of diseased versus normal specimens [33-36]. LCM is a one-step microdissection technique for acquiring cells from specific regions of tissue sections under direct visualization, while maintaining structural integrity and morphology. Therefore, it allows for in situ studies that correlate to in vivo events and decreases the likelihood of generating in vitro artifacts by minimizing tissue manipulation during sample acquisition [37]. Early LCM studies have shown that the LCM process itself does not alter samples [37]. This technique has mostly been used in studies investigating cancers in various tissues like the human brain [38], cervix [33], kidney [33], bladder [39], prostate [40], and colon [41], a small number of which have followed LCM with protein analysis rather than DNA/RNA analysis. Studies also identified the spatial distribution of modified and unmodified proteins using two-dimensional electrophoresis (2-DE) of microdissected tissue regions [42-44]. Preliminary proteomic studies have shown compatibility of LCM with two-dimensional PAGE (2-D PAGE) to examine protein profiles of selected tissues, and often noted enrichment of some proteins compared to analyses of whole tissues [33,37,44]. However, crystallins and their modifications have not been analyzed using LCM, although PTMs of human αA-crystallin alone have been successfully studied using manual microdissection [45]. The study correlated increased truncation and modification of αA-crystallin with lens fiber cell age and depth within the lens.

Development of a multiplexing fluorescent electrophoresis method, 2D-DIGE, has been an advancement in 2-D PAGE [40,46] that allows labeling of 2–3 samples with different dyes, including an internal control, which is a pool of all samples that were individually labeled. All samples can be run on a single 2-D gel to minimize gel-to-gel variation and the number of gels required for one experiment. The ability to multiplex different sulfonated indocyanine dye (CyDye) DIGE Fluor minimal dye-labeled samples on the same gel means that the different samples will be subject to exactly the same 1st and 2nd dimension running conditions. Consequently, the same protein labeled with any of the CyDye DIGE Fluor minimal dyes and separated on the same gel will migrate to the same position on the 2D-gel and overlay. Again, this limits experimental variation and ensures accurate within-gel matching. Recent studies in cancer proteomics have applied 2D-DIGE analysis to LCM-acquired samples and have found it to be a powerful tool suitable for mass spectrometric protein identification [35].

To avoid the pitfalls of studying PTMs in human crystallins using whole lens homogenates, the present study used both LCM and 2D-DIGE to identify the regional distribution of crystallins and their PTMs in cortex and nucleus with age of a single lens. The present study reports a novel finding that truncation of crystallins begins in the cortical region and progressively extends to the nuclear region, while crystallin aggregation mainly occurs in the nucleus. These changes in crystallins represent those that occur during fiber cell migration from the periphery (cortex) to the central region (nucleus) with aging.

## Methods

### Tissue preparation and sectioning

Intact frozen normal human donor lenses, stored at −80 °C following excision from globes, were provided by Dr. Christine Curcio (UAB Department of Ophthalmology, Birmingham, AL). The lenses did not show any opacity and therefore were considered normal. Lenses were collected and transported on dry ice, then stored at −80 °C before use. Two lenses, one from a 65- and the other from 69-year-old donor, were thawed, embedded directly in optimum cutting temperature (OCT) compound (Tissue-Tek®; Sakura Finetek, Torrance, CA) at −20 °C, and tissue blocks were cut into 12 μm sections using a Shandon Cryotome E (ThermoFisher Scientific, Kalamazoo, MI). The multiple sections acquired were from anterior, equatorial, and posterior regions of the lenses. Individual sections were mounted onto PALM® polyethylene naphthalate (PEN) membrane slides (Carl Zeiss MicroImaging GmbH, Germany) and/or Fisherbrand Superfrost glass slides (Fisher Scientific, Pittsburgh, PA). Slides were stored at −80 °C before performing laser capture microdissection (LCM).

### Laser capture microdissection

Just before microdissection, slides containing frozen sections were quick-thawed to room temperature, then fixed and dehydrated using a graded series of alcohols and cleared in xylene as follows: 70% ethanol (15 s), 95% (15 s), 100% (15 s), and xylene (5 min). The sections were then air-dried.

**Figure 1:**
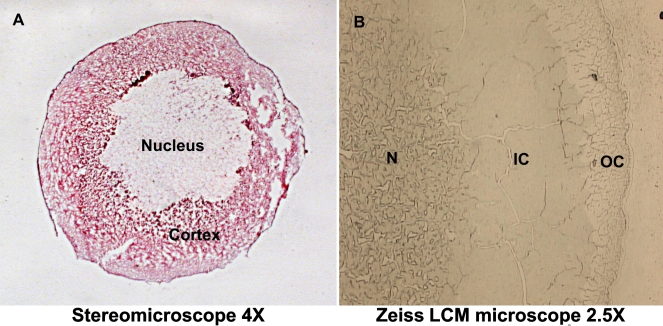
Tissue section of a human lens. **A**: Whole tissue section (12 μm) of 69-year-old lens stained with hematoxylin and eosin, as seen under a stereomicroscope with 4× magnification. All three major regions are present in this section, however the nucleus stained more faintly because of possible increased hydrophobicity of the proteins in this region. **B**: Unstained section showing three major lenticular regions: Nucleus (N), Inner Cortex (IC), and Outer Cortex (OC), as seen during LCM with the Zeiss/PALM Microbeam microscope at its lowest magnification of 2.5×.

Cells from either three or two major lenticular regions – outer cortex, inner cortex, and nucleus, or outer and inner cortex combined and nucleus alone (Figure 1) – were microdissected (Zeiss/PALM Microbeam; Carl Zeiss) by outlining regions of interest and pressure catapulting selected objects using the Auto LPC software function. Pulses of a high quality UV-A laser beam (<1 μm spot size) cut and eject/catapult selected objects into individual PALM® adhesive cap microfuge tubes (Carl Zeiss). Microfuge tubes containing captured tissue were transported on dry ice and stored at −80 °C before processing.

### Protein extraction

Following microdissection, samples were processed for desired types of electrophoretic analysis. For SDS–PAGE, captured tissue from each of three regions, stated above, was individually solubilized in Buffer A (50 mM Tris-HCl, pH 7.8, 1 mM phenylmethylsulphonyl fluoride [PMSF], 1 mM iodoacetamide, 1 mM dithiothreitol [DTT]), then microcentrifuged (13,400× g, 10 min, 4 °C) (Eppendorf Centrifuge 5415R; Eppendorf of North America, Westbury, NY). Protein concentration was measured by absorbance at 280 nm using a NanoDrop® ND-1000 Full Spectrum (200–750 nm) Spectrophotometer (NanoDrop Technologies Inc., Wilmington, DE).

For 2D-DIGE and 2-D PAGE analyses, captured tissue was extracted using extraction buffer (EB; 7.8 M urea, 2.2 M thiourea, 2.2% [v/v] pharmalyte, pH 3–10 [Amersham, Hercules, CA], 1.1% [v/v] protease inhibitor cocktail [Sigma Aldrich, St. Louis, MO]), followed by addition of detergent (40% [v/v] -[(3-Cholamidopropyl)dimethylammonio]-1-propansulfonate [CHAPS]). The buffer solubilized both soluble and insoluble proteins from lens sections. Samples were then reduced with 5 mM tributylphosphine (TBP; Sigma) and alkylated with 20 mM 4-vinylpyridine (4-VP; Sigma). Pharmalytes were removed from 2D-DIGE samples by exchange of EB with DIGE exchange buffer (DExB; 7 M urea, 2 M thiourea, 30 mM Tris, 4% [w/v] CHAPS) using a Millipore Centrifugal filter with a membrane cutoff of 10 kDa. Protein was then quantified using a 2-D Quant Kit (GE Healthcare Biosciences, Piscataway, NJ) and labeled as described below.

### SDS–PAGE analysis

Equal amounts of protein (10 μg) from three lenticular regions (outer cortical, inner cortical, and nuclear regions) were analyzed by one-dimensional sodium dodecyl sulfate – PAGE (SDS–PAGE) [47], and gels were stained with Coomassie blue R250 stain (Fisher). Individual protein bands were excised and processed for Matrix-assisted Laser Desorption Ionization-Time of Flight (MALDI-TOF) mass spectrometric analysis. Samples were destained using three washes of 25 mM ammonium bicarbonate (ABC)/50% acetonitrile and were digested with trypsin (12.5 ng/μl) plus 50 mM ABC for 16 h at 37 °C. Peptides were extracted with two washes in a 50/50 solution of 5% formic acid and 100% acetonitrile, dried down in a Savant Speed Vac Concentrator (Forma Scientific, Millford, MA), and resuspended in 10 μl of 0.1% formic acid (FA). Peptides were desalted using C18 Zip Tips^™^ (Millipore, Bradford, MA) and further analyzed by MALDI-TOF mass spectrometry.

### 2D-DIGE analysis

CyDye DIGE Fluor minimal dyes are very useful for multicolor analysis and also specially developed to be size and charge matched specifically for Ettan DIGE Systems (GE Healthcare). The protein samples and the internal standard are each labeled with a different CyDye DIGE Fluor minimal dye, after which these labeled samples are combined, run on an isoelectric focusing (IEF) gel in the first dimension, and separated by SDS–PAGE in the second dimension (GE Healthcare). Proteins from cortical and nuclear regions were separated by 2D-DIGE. First dimension IEF was performed by active rehydration of an 11 cm immobilized pH gradient gel (IPG) strip, pH 5–8. This was performed at 500V with rehydration buffer (7M urea, 2M thiourea, 2% [v/v] pharmalyte, pH 3–10) and using a PROTEAN IEF System (Bio-Rad, Hercules, CA). An internal standard, containing pooled cortical and nuclear proteins was labeled with Cy2, cortical proteins from both outer and inner cortex were labeled with Cy3, and nuclear proteins were labeled with Cy5. Technical replicates, created by dye-swapping the labeled samples, were also run. This was followed by desalting at 300 V for 4 h and focusing at 3,500 V for 9 h. Focused strips were equilibrated for 15 min before running the second dimension with equilibration buffer containing 6M urea, 50 mM Tris-HCl, pH 8.8, 2% SDS, 30% glycerol, and 0.01% bromophenol blue.

In the second dimension, proteins were separated by SDS–PAGE using 23×20 cm 15% polyacrylamide gels. IPG strips were immobilized at the top of second dimension slab gels using 1% low-melt agarose in 3× running buffer containing bromophenol blue (24 mM Tris-HCl, 192 mM glycine, 1% SDS, 0.01% bromophenol blue), which was added as a tracking dye. Second dimension gels were run for 1 h at 1 W/gel, and then at 15 W/gel for 6 h. Gels were post-stained with SYPRO Ruby protein gel stain (Bio-Rad) and imaged with a Typhoon 9400^™^ scanner (GE Healthcare). Image analysis was done using DeCyder^™^ v6.5 software (GE Healthcare) accompanying the Typhoon^™^ 9400 scanner (GE Healthcare).

Gels were secondarily post-stained with Coomassie blue stain (Fisher) to increase visibility for manual spot picking. Individual excised spots were destained, trypsin-digested, and extracted similarly to samples processed for MALDI-TOF analysis, as described above. Supernatants were also dried down in a speed vac (Forma Scientific) and resuspended in 10 μl of 0.1% formic acid. Peptides were analyzed by Electrospray Ionization Quadrupole Linear Ion-Trap Liquid Chromatography Mass Spectrometry (ESI-QTRAP LC-MS/MS).

### 2-D PAGE analysis

Proteins from the nuclear region alone were separated by 2-D PAGE. Similarly to samples from 2D-DIGE, the first dimension IEF and second dimension SDS–PAGE were performed using an 11 cm IPG strip, pH 5–8, but without CyDye labeling. The gel was post-stained with Coomassie blue stain (Fisher) and imaged.

### Identification of lens proteins

MALDI-TOF and ESI-QTRAP LC-MS/MS analyses were performed at the Comprehensive Cancer Center Mass Spectrometry Shared Facility of the University of Alabama at Birmingham (Brimingham, AL). Individual protein bands or spots were manually excised from SDS–PAGE, 2D-PAGE, and 2D-DIGE gels that were post-stained with Coomassie blue R250 (Fisher) for identification purposes, and subjected to processing for mass spectrometric analysis. The polyacrylamide pieces containing individual spots were destained with three consecutive washes with a mixture of 50% 25 mM ammonium bicarbonate/50% acetonitrile for 30 min. Next, the samples were washed for 10 min with 25 mM ammonium bicarbonate before digestion with trypsin (12 ng/μl; sequencing grade from Roche) for 16 h at 37 °C. Peptide solutions were then extracted using 100 μl of a 50/50 solution of 5% formic acid and acetonitrile for 30 min. Supernatants were collected and dried to dryness in a Savant Speed Vac Concentrator (Forma Scientific). Samples were resuspended in 10 μl of 0.1% formic acid. Tryptic peptides extracted from the SDS–PAGE gel bands were mixed in 1/10 dilutions with a mixture of α-cyano-4-hydroxycinnamic acid (CHCA; 5 mg/ml; Sigma) matrix dissolved in acetonitrile: 0.1% trifluroacetic acid (1:1). C18 Zip Tips (Millipore) were used to desalt peptide mixtures before applying samples to the MALDI-TOF 96×2 well target plates. Samples were applied to a MALDI-TOF 96-well target plate (Applied Biosystems, Foster City, CA), air-dried, and analyzed using Voyager DE-Pro scanning from 900 to 4000 m/z in positive ion mode (Applied Biosystems). Spectra were then analyzed using Voyager Explorer software, and peptide masses were entered into the Matrix Science: MASCOT database for identification of peptides. The MALDI-TOF identity of proteins was established by using the NCBInr database from Matrix Science.

Tryptic peptides extracted from 2D-DIGE and 2-D PAGE gel spots and bands were analyzed using an ABI 4000 QTRAP LC-MS/MS Mass Spectrophotometer (Applied Biosystems). Five microliters per sample of trypsin digested peptides were injected into the spectrophotometer and eluted off of a capillary C-18 reverse-phase column using an H_2_O/acetonitrile gradient, then fragmented in the QTRAP. Columns were washed between sample analyses. For identification, the resulting spectra were processed using Analyst Software (Applied Biosystems), and all data were subjected to a MASCOT server peptide-sequencing search against all known mammalian proteins found in the NCBI and Unihuman2 protein databases. Peptide identifications from the MASCOT search were accepted for peptides scoring 40 and above within each protein identification of a given spot. Identifications were performed, and scoring criteria were set with the help of a qualified mass spectrometry specialist in the Comprehensive Cancer Center - Mass Spectrometry and Proteomics shared facility at the University of Alabama at Birmingham (Birmingham, AL).

## Results

### Laser microdissection and protein identification following SDS–PAGE

To determine the crystallin profile of a normal human aging lens using LCM and 2D-DIGE, it was necessary to first acquire intact sections of appropriate thickness from the human lenses (Figure 1). Acquiring intact 12 μm sections was technically difficult because the water content and high protein concentration of the lens make the tissue harder and brittle when frozen. During tissue embedding, OCT does not infiltrate the tissue and become miscible with the water, therefore some cracking was visible during sectioning. After considerable trial and error, the technique was standardized and 12 μm intact sections were recovered (see Methods for details). These lens sections were used for LCM initially to recover tissue constituting the outer cortical, inner cortical and nuclear regions. Later, tissue from outer and inner cortical regions were combined and referred to as the cortical region (see below). This pooling was done because the GE Healthcare 2D-DIGE protocol suggested that approximately 40 μg of total protein was needed to efficiently label them with CyDyes. An initial extraction of proteins from a single 12 μm section of a 65 year-old normal human lens was performed to estimate how many lens sections were needed to acquire sufficient quantity of protein from each of the three lens regions (i.e., outer cortical, inner cortical and nuclear regions). As shown in Table 1, LCM of the 12 μm sections from 65 year-old or 69 year-old lenses yielded protein ranging from 0.6 to 2 μg/section from the outer cortical region, 13–15 μg/section from the inner cortical region, and 12–15 μg/section from the nuclear region.

**Table 1:** Quantities of protein recovered by LCM from outer cortex, inner cortex, and nuclear regions in 12 μm sections of human lens.

**Lenses**	**Number of sections**	**Outer cortex total protein (μg)**	**Inner cortex total protein (μg)**	**Nucleus total protein (μg)**
65-year-old	6	12	78	70
69-year-old	12	7	182	182

**Figure 2:**
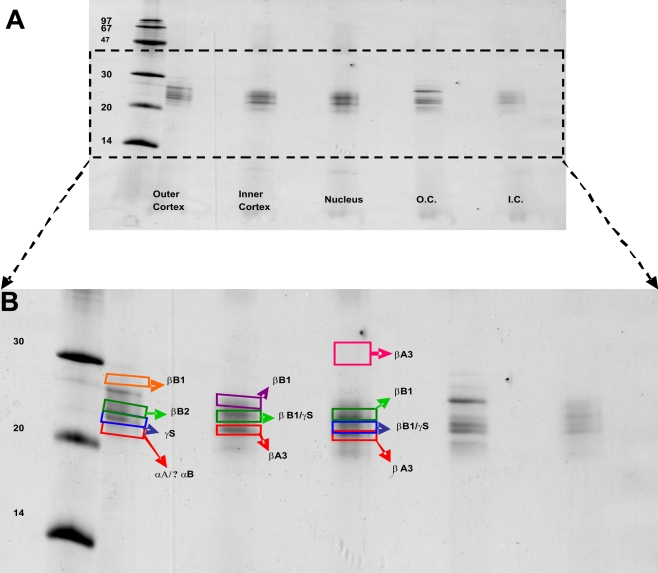
Separation of 65-year-old human lens proteins using 15% polyacrylamide gel by SDS–PAGE analysis and identification of excised bands by MALDI-TOF mass spectrometry. **A**: SDS–PAGE image of proteins in three lenticular regions with Coomassie Blue R250 staining showing mostly LMW species. Samples from the outer and inner cortices were repeated in the last two lanes on the right side of the gel. **B**: Expanded image seen in **A**. Boxes outline the bands excised for mass spectrometric analysis and crystallin identifications of excised bands were based on MALDI-TOF data.

MALDI-TOF analysis of individual protein bands from the SDS-gel revealed the presence of αA- and αB-crystallins (spectral data not shown) in the outer cortical region, βA3-crystallin in both the inner cortical and nuclear regions, and a truncated βA3 species in the nuclear region (Figure 2). Although initial findings spurred further analysis, additional preliminary experiments proved that it was difficult to obtain sufficient amounts of protein from the outer cortical region (Table 1).

In the lens sections, the cortical tissue (both inner and outer cortex) constitutes ~30%–40% of the lens tissue section, whereas the nuclear region constitutes ~60%–70% (Figure 1). Because of low protein recovery, the tissues from both inner and outer cortical regions, although captured separately, were pooled and referred to as the cortical region in subsequent experiments.

### Detection of proteins in cortical and nuclear regions

**Figure 3:**
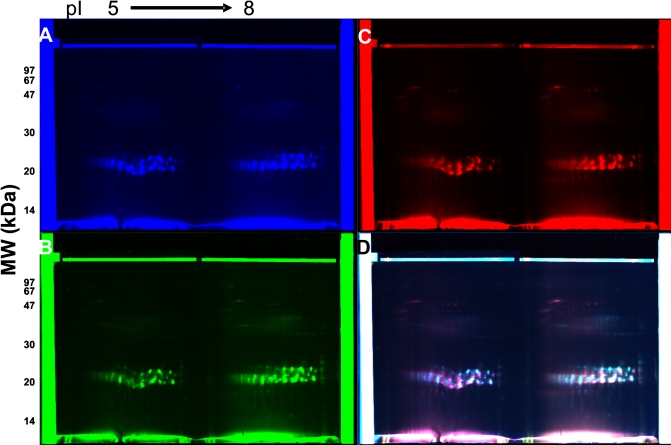
Typhoon-scanned fluorescence images from a 2D-DIGE of a 69-year-old human lens. Equal concentrations of protein (20 μg) from the cortex and nucleus were labeled with different fluorescent dyes and analyzed by 2D-DIGE. **A**: Internal standard labeled with Cy2 (Ex 488 nm, Em 520 nm). **B**: Cortical proteins (outer and inner cortex pooled) labeled with Cy3 (Ex 532 nm, Em 580 nm). **C**: Nuclear proteins labeled with Cy5 (Ex 633 nm, Em 670 nm). **D**: Overlay of **A**, **B**, and **C**. Spots fluorescing white are protein species present in both cortex and nucleus, while those fluorescing blue and red are localized to the corresponding regions as shown in **B** and **C**, respectively.

Tissue samples, selectively collected by LCM from the cortical and nuclear regions of a 69-year-old normal human lens, were used for analysis by 2D-DIGE. Following protein extraction (see Methods), equal amounts of protein (20 μg) from each region, along with an internal standard (pooled proteins from the two regions), were labeled with different fluorescent dyes (Cy2 – internal standard, Cy3 – cortical region, Cy5 – nuclear region). Figure 3A shows the internal standard labeled with Cy2 (Excitation at 488 nm, Emission at 520 nm), 3B shows the cortical proteins labeled with Cy3 (Excitation at 532 nm, Emission at 580 nm), and 3C shows nuclear proteins labeled with Cy5 (Excitation at 633 nm, Emission at 670 nm). Figure 3D is an overlay of the images and identifies white fluorescing protein spots common to both cortical and nuclear regions, whereas red and blue fluorescing spots were from nuclear and cortical regions, respectively.

### Protein and PTM identification in 2-D gel electrophoresis

**Figure 4:**
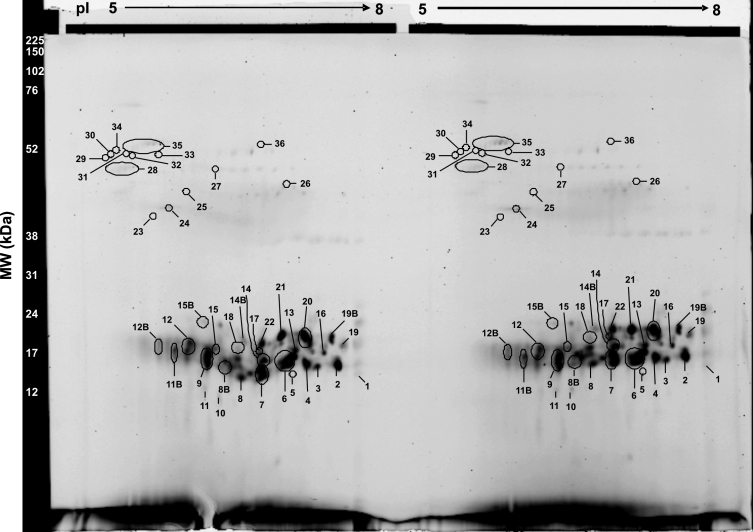
Coomassie blue-stained gel from 2D-DIGE of 69-year-old human lens. The gel was stained with Coomassie blue R250 for the purpose of manually picking the individually labeled spots (circled and numbered in the figure) for identification by ESI-QTRAP LC/MS/MS. The gels in this figure are technical replicates, so corresponding spots were picked and pooled for identification.

To identify the crystallin species present in individual spots of cortical and nuclear regions, the 2D-DIGE gel was secondarily stained with Coomassie blue (Figure 4). Each labeled spot was manually excised and processed for analysis by Q-TRAP LC-MS/MS (see Methods). The mass spectrometric data were tabulated and spots were distributed into the following three categories based on their molecular weights (M_r_): (1) Crystallin fragments (M_r_ <20 kDa), (2) Intact crystallins (M_r_ 20–35 kDa), and (3) High molecular weight (HMW) proteins (M_r_ >35 kDa; Table 2). A total of 36 spots were observed with identification of spots 1–11B as crystallin fragments, spots 12–22 as intact crystallins, and spots 23–36 as HMW proteins (Figure 4 and Table 2). Table 2 provides a summary of crystallin species present in each excised spot and their relative localizations in the two lenticular regions, while Appendix 1 shows the amino acid sequences of their tryptic peptides as identified by ESI-QTRAP LC-MS/MS. Spots 1–11B contained tryptic fragments of αA-, αB-, βA3-, βA4-, βB1-, βB2-, βB3-, γB-, γC-, γD-, and γS-crystallin (Table 2). Taken together, the results shown in Table 2 and Appendix 1 suggested that truncation of α-, β-, and γ-crystallins begins in the cortical region. Spots 12–22 contained tryptic peptides of αA-, αB-, βA3-, βA4-, βB1-, βB2-, βB3-, γB-, γC-, γD-, and γS-crystallin (Table 2), but from the tryptic peptide sequences of spots 12–22, it was unclear whether these were intact crystallins. Additionally, DeCyder analysis software that accompanies the Typhoon scanner used to image the 2D-DIGE gels was also applied, with the help of a qualified specialist in the Proteomics Core facility at UAB. Based on technical replicates and software analysis, Table 3 identifies relative fold differences in volume ratios of overlapping spots, in both cortical and nuclear regions, in the 2D-DIGE profile of a 69-year-old lens (Figure 3). DeCyder software analysis selectively identified only 22 out of 36 spots as statistically significant. Therefore, the values listed in the last column showed statistically significant differences (p<0.05) determined by the ratio of intensity of one fluorophore to another in overlapping spots (Table 3). DeCyder Differential In-gel Analysis (DIA) co-detects three images created from the internal standard and the two samples, and determines differences in the intensity of spots within the gel that are matched across all images (Figure 3). Significance was based on parameters for Student’s *t*-test entered into the software. As seen in Table 3, among the crystallin fragments (M_r_ <20 kDa), spot 6 (containing fragments of αA-, αB-, βA3-, γD-, and γS-crystallin), spot 8B (containing fragments of αB- and βA3-crystallin), and spot 11 (containing fragments of αA-, αB-, and βA4-crystallin) showed a 1.2–3 fold greater abundance in the nuclear region compared to the cortical region. In contrast, spot 11B (containing fragments of αA-, αB-, and βA4-crystallin) showed roughly twofold greater abundance in the cortical region compared to the nuclear region. A similar analysis of intact crystallins with M_r_ 20–35 kDa showed differential distribution in the cortical and nuclear regions. Spot 12 (containing αA- and γS-crystallin), spot 12B (containing αA-, αB-, βA3-, βB1-, βB2-, γC-, and γS-crystallin), spot 14B (containing βB2-crystallin), and spot 15B (containing βA3-, βA4-, βB2-, and γS-crystallin) showed about twofold greater abundance in the cortical region compared to the nuclear region. Spots 23–36, with M_r_ >35 kDa, represented crystallin multimers and showed greater abundance in the nuclear region compared to the cortical region.

**Table 2:** Summary of crystallin species in spots from Coomassie-stained 2D-DIGE gel of 69-year-old human lens identified by ESI-QTRAP LC-MS/MS.

**Spot number**	**Identification by Q-TRAP**	**M_r_ (kDa)**	**Localization**
1	γD	<20	N>C
2	αA chain, αB	<20	N<C
3	αA chain, αB, βA3, βB3	<20	N<C
4	αB, βA3, βB1, γD	<20	N=C
5	αB, βA3, γD	<20	N>C
6	αA, αB, βA3, γD, γS	<20	N=C
7	αA, αB,βA3, βB1, βB2, γB, γS	<20	N=C
8	αB, βA3	<20	N=C
8B	αB, βA3	<20	N>C
9	αB, βA3, βA4	<20	N<C
10	αA,, αB, βA3, βA4	<20	N<C
11	αA, βA3, βA4	<20	N>C
11B	αA,, αB, βA4	<20	N<C
12	αA, γS	20 - 35	N<C
12B	αA, αB, βA3, βB1, βB2, γC, γS	20 - 35	N<C
13	αA, βA4, βB1, βB2, γS	20 - 35	N<C
14	βA3, βB1, γS	20 - 35	N=C
14B	βB2	20 - 35	N<C
15	βA3, βB1, βB2, βA4, γS	20 - 35	N=C
15B	βA3, βA4, βB2, γS	20 - 35	N<C
16	αA, βB1, βB2	20 - 35	N<C
17	truncated βB1 chain, βB1, βB2	20 - 35	N=C
18	βB1, βB2	20 - 35	N>C
19	αA chain, βB1, βB2	20 - 35	N>C
19B	αA, αB, βA3, βB2, βB4	20 - 35	N<C
20	αB, βA3, βB1, βB2, γS	20 - 35	N=C
21	αA, αB, βA3, βB1, βB2	20 - 35	N<C
22	αA	20 - 35	N<C
23	NI	>35	N>C
24	NI	>35	N>C
25	NI	>35	N>C
26	NI	>35	N<C
27	NI	>35	N>C
28	CP49	>35	N>C
29	NI	>35	N>C
30	NI	>35	N>C
31	NI	>35	N>C
32	NI	>35	N>C
33	NI	>35	N>C
34	NI	>35	N>C
35	αA, Filensin	>35	N>C
36	NI	>35	N>C

NI=Not Identified by mass spectrometry. Data from the “Localization” column was based on DIGE analysis.

**Table 3:** DeCyder software analysis highlighted statistically significant spots, based on technical replicates, differentially expressed in nuclear and cortical regions (p<0.05) from the 2D-DIGE gel of a 69 year-old lens (Figure 3) and crystallin species were then identified by ESI-QTRAP LC-MS/MS (Appendix 1).

**Spot number**	**Crystallin species**	***M*_r_ (kDa)**	**Nucleus/cortex**
6	αA chain, αB, βA3, γD, γS	<20	1.24/1
8B	αB, βA3	<20	2.08/1
11	αA, βA3, βA4	<20	3.14/1
11B	αA, αB chain, βA4	<20	−1.61/1
12	αA, γS	20 - 35	−1.97/1
12B	αA, αB chain, βA3, βB1, βB2, γC, γS	20 - 35	−1.51/1
14B	βB2	20 - 35	−2.06/1
15B	βA3, βA4, βB2, γS	20 - 35	−2.21/1
17	Truncated βB1, βB2	20 - 35	2.01/1
23	NI	>35	3.00/1
24	NI	>35	2.17/1
25	NI	>35	2.84/1
26	NI	>35	−3.14/1
27	NI	>35	1.96/1
29	NI	>35	2.49/1
30	NI	>35	3.09/1
31	NI	>35	2.18/1
32	NI	>35	3.42/1
33	NI	>35	3.58/1
34	NI	>35	3.65/1
35	αA, filensin	>35	3.91/1
36	NI	>35	3.40/1

Values given in the last column represent fold differences in abundance between nuclear and cortical regions, with the sign of the fold value representing up- or down-regulation. NI=Not identified by mass spectrometry because of low concentrations, although DeCyder determined relative abundance.

**Figure 5:**
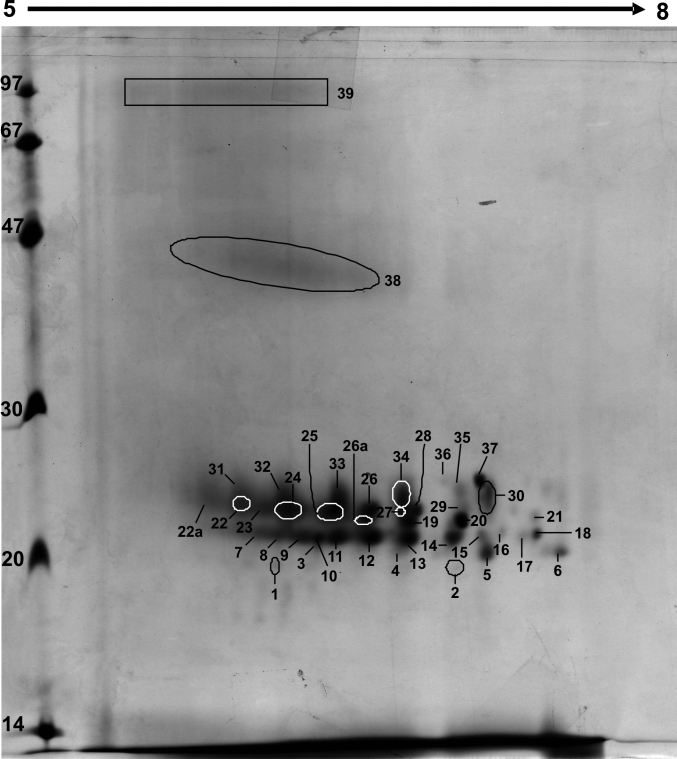
Standard 2D gel of a 69-year-old human lens. IEF was done in the first dimension using an 11 cm IPG strip, pH 5–8, followed by SDS–PAGE using a 15% polyacrylamide gel in the second dimension. The gel was stained with Coomassie blue R250, and shows a profile similar to 2D-DIGE gels of the same tissue. However, HMW (>35 kDa) aggregates are not distinguished as individual spots, rather they appeared as non-descript bands.

A major problem encountered in the 2D-DIGE experiment was identification of crystallins that existed as HMW protein spots with M_r_ >35 kDa. ESI-QTRAP LC-MS/MS analysis successfully identified both spot 28 containing CP49 and spot 35 that contained αA-crystallin and filensin (Tables 2 and 3), with spot 35 having almost a fourfold greater abundance in the nuclear region compared to the cortical region (Table 3). Identification of HMW protein spots 23–27, 29–34, and 36 failed because of their low protein concentrations (Figure 4). Therefore, protein yield was increased by extraction from a greater number of lens sections, and specifically from the nuclear region. Tissue from the nuclear region of 48 sections (12 μm) of a 69-year-old lens was captured by LCM and processed for standard 2D-gel electrophoresis. The 2D gel protein profile exhibited a pattern similar to that of the 2D-DIGE gel (compare protein profiles of gels in Figure 4 and Figure 5). However, unlike the 2D-DIGE gel, 39 spots were resolved and the HMW proteins appeared as non-descript bands that were labeled as numbers 38 and 39 (Figure 5). The appearance of non-descript bands was likely because the majority of proteins in the nuclear region are highly modified and cross-linked. Results showed that band 38 contained a mixture of αA-, αB-, βA3-, βA4-, βB1-, γB-, γC-, γD-, and γS-crystallins and CP49, whereas band 39 was a mixture of αA-, αB-, βA3-, βA4-, βB1-, βB2-, γB-, γC-, γD-, and γS-crystallin and both filensin and CP49 (Table 4). Table 5 shows the amino acid sequences of tryptic peptides of crystallin species identified in bands 38 and 39 of Figure 5.

**Table 4:** Identification of crystallins present in the HMW bands of a 69-year-old lens following 2D-gel electrophoresis (Figure 5).

**Band number**	**Identification by QTRAP**
38	αA-, αB-, βA3-, βA4-, βB1-, γB-, γC-, γD-, γS-crystallin
	filensin
39	αA-, αB-, βA3-, βA4-, βB1-, βB2-, γB-, γC-, γD-, γS-crystallin
	CP49, filensin

**Table 5:** Crystallin species, sequences, and modifications in HMW bands from 2D gel of the nuclear region of a 69-year-old human lens identified by ESI-QTRAP LC-MS/MS (Figure 5).

**Band number**	**α-Crystallin**	**β- and γ-Crystallin**	**CP49**	**Filensin**
38	αA: #1 – 11 MDVTIQHPWFK [Acet(N-term);Oxi(M)]	βA3: #33 – 44 ITIYDQENFQGK [Deam(NQ)]		#78 – 90 LGELAGPEDALAR
	αA: #13 – 21 TLGPFYPSR	βA3: #33 – 45 ITIYDQENFQGKR [Deam(NQ)]		#99 – 106 VRDLEAER
	αA: #55 – 65 TVLDSGISEVR	βA3: #96 – 109 WDAWSGSNAYHIER		
	αA: #79 – 88 HFSPEDLTVK	βA3: #126 – 137 MTIFEKENFIGR Eth(N-term);Oxi(M)]		
	αA: #89 – 99 VQDDFVEIHGK	βA3: #197 – 211 EWGSHAQTSQIQSIR		
	αA: #146 – 157 IQTGLDATHAER	βA4: #14 – 25 MVVWDEDGFQGR [Oxi(M)]		
	αB: #12 – 22 RPFFPFHSPSR	βA4: #107 – 118 LTIFEQENFLGK [Deam(NQ)]		
		βA4: #178 – 192 EWGSHAPTFQVQSIR		
		βB1: #51 – 60 AAELPPGNYR		
		βB1: #61 – 72 LVVFELENFQGR		
		βB1: #73 – 86 RAEFSGECSNLADR [Phos(STY)]		
		βB1: #74 – 86 AEFSGECSNLADR		
		βB1: #111 – 118 GEMFILEK		
		βB1: #124 – 132 WNTWSSSYR [Deam(NQ)]		
		βB1: #136 – 143 LMSFRPIK		
		βB1: #151 – 160 ISLFEGANFK		
		βB1: #188 – 202 VSSGTWVGYQYPGYR [Oxi(HW)]		
		βB1: #203 – 214 GYQYLLEPGDFR		
		γB: #4 – 10 ITFYEDR		
		γB: #60 – 77 RGEYPDYQQWMGLSDSIR		
		γB: #155 – 164 FLDWGAPNAK		
		γC: #4 – 10 ITFYEDR		
		γC: #60 – 77 RGEYPDYQQWMGLSDSIR		
		γC: #116 – 122 FHLSEIR		
		γD: #2 – 10 GKITLYEDR		
		γD: #4 – 10 ITLYEDR		
		γD: #60 – 77 RGDYADHQQWMGLSDSVR		
		γD: #61 – 77 GDYADHQQWMGLSDSVR [Oxi(M)]		
		γD: #81 – 89 LIPHSGSHR		
		γD: #143 – 152 QYLLMPGDYR [Oxi(M)]		
		γD: #154 – 163 YQDWGATNAR [Oxi(HW)]		
		γS: #8 – 14 ITFYEDK [Eth(K)]		
		γS: #8 – 19 ITFYEDKNFQGR [Cam(N-term);Deam(NQ)]		
		γS: #42 – 52 VEGGTWAVYER		
		γS: #73 – 79 WMGLNDR		
		γS: #85 – 95 AVHLPSGGQYK [Deam(NQ)]		
		γS: #147 – 154 GRQYLLDK		
		γS: #149 – 155 QYLLDKK		
		γS: #159 – 166 KPIDWGAA		
		γS: #159 – 174 KPIDWGAASPAVQSFR [Deam(NQ);Oxi(HW)]		
		γS: #159 – 175 KPIDWGAASPAVQSFRR		
39	αA: #1 – 11 MDVTIQHPWFK	βA3: #33 – 44 ITIYDQENFQGK [Deam(NQ)]	#77 – 89 ALGISSVFLQGLR	#78 – 90 LGELAGPEDALAR
	αA: #1 – 12 MDVTIQHPWFKR	βA3: #96 – 109 WDAWSGSNAYHIER [Deam(NQ)]		#96 – 106 VRDLEAER
	αA: #13 – 21 TLGPFYPSR	βA3: #126 – 137 MTIFEKENFIGR [DeamNQ);Oxi(M)]		
	αA: #55 – 65 TVLDSGISEVR	βA3: #197 – 211 EWGSHAQTSQIQSIR		
	αA: #79 – 88 HFSPEDLTVK	βA4: #14 – 25 MVVWDEDGFQFR [Oxi(M)]		
	αA: #89 – 99 VQDDFVEIHGK	βA4: #107 – 118 LTIFEQENFLGK		
	αA: #146 – 157 IQTGLDATHAER	βB1: #51 – 60 AAELPPGNYR		
	αB: #57 – 69 APSWFDTGLSEMR [Oxi(M)]	βB1: #61 – 72 LVVFELENFQGR		
	αB: #73 – 82 DRFSVNLDVK	βB1: #73 – 86 RAEFSGECSNLADR [Phos(STY)]		
	αB: #83 – 92 HFSPEELKVK	βB1: #111 – 118 GEMFILEK		
	αB: #93 – 103 VLGDVIEVHGK	βB1: #111 – 123 GEMFILEKGEYPR		
		βB1: #124 – 132 WNTWSSSYR [Deam(NQ)]		
		βB1: #133 – 143 SDRLMSFRPIK		
		βB1: #136 – 143 LMSFRPIK		
		βB1: #136 – 150 LMSFRPIKMDAQEHK		
		βB1: #151 – 160 ISLFEGANFK		
		βB1: #171 – 182 APSLWVYGFSDR		
		βB1: #188 – 202 VSSGTWVGYQYPGYR [Deam(NQ);Oxi(HW)]		
		βB1: #203 – 214 GYQYLLEPGDFR		
		βB2: #109 – 120 IILYENPNFTGK		
		βB2: #146 – 160 VQSGTWVGYQYPGYR		
		βB2: #161 – 168 GLQYLLEK		
		βB2: #169 – 188 GDYKDSSDFGAPHPQVQSVR		
		βB2: #190 – 198 IRDMQWHQR		
		γB: #4 – 10 ITFYEDR		
		γB: #155 – 164 FLDWGAPNAK		
		γC: #4 – 10 ITFYEDR		
		γC: #116 – 122 FHLSEIR		
		γD: #4 – 10 ITLYEDR		
		γD: #143 – 152 QYLLMPGDYR		
		γD: #154 – 163 YQDWGATNAR		
		γS: #8 – 14 ITFYEDK [Eth(K)]		
		γS: #8 – 19 ITFYEDKNFQGR		
		γS: #73 – 79 WMGLNDR		
		γS: #85 – 95 AVHLPSGGQYK		
		γS: #159 – 174 KPIDWGAASPAVQSFR [Kyn(W);Oxi(HW)]		
		γS: #159 – 175 KPIDWGAASPAVQSFRR		

Underlined amino acids mark sites of modification, and modifications are shown in brackets. Abbreviations Used: Acet – Acetylation; Cam – Carbamylation; Deam – Deamidation; Eth – Ethylation; Fkyn – Formylkynurenin=Double oxidation of Trp; Kyn – Kynurenin=Triple oxidation of Trp; Meth – Methylation; Oxi – Oxidation=Single oxidation; Phos – Phosphorylation; Sulph - Sulphone.

Q-TRAP analysis also identified several PTMs in crystallins including: αA-, αB-, βA3-, βA4-, βB1-, βB2-, γD-, and γS-crystallins, as well as CP49, which are summarized in Table 6. Table 6 and Appendix 1 show the amino acid sequences, specific modification sites within these tryptic sequences (see underlined amino acids), and PTMs (see brackets) of such modified proteins as those described. PTMs found in αA-crystallin species were present in both the cortical and nuclear regions. The same was true for αB-crystallin, except for a single oxidized M68 residue that was only present in the HMW region and, therefore, only found in the nuclear region. βA3-crystallin exhibited modifications that were found in both cortical and nuclear regions, however, deamidation of residues Q38, N40, and Q42 was seen only in the HMW proteins of the nuclear region. Among the crystallins, the fewest modifications were seen in βA4-crystallin, which contained only seven PTMs (i.e., two deamidations of residue N114, and five oxidations of residue M14). Unlike the PTMs seen in the α-crystallins, modifications of βB1-crystallin mostly occurred in the HMW proteins of the nuclear region, while only two modifications, deamidations of N68 and Q236, occurred in the lower molecular weight (LMW) crystallins (<35 kDa). All the modified species of βB2-crystallin were found only in LMW proteins and the majority of the PTMs were deamidations, followed by oxidations, and ethylations. Among the γ-crystallins, γS-crystallin showed the most modifications. Oxidations of residues M70, M147, and W157 of γD-crystallin were all present only in HMW proteins. In contrast, several modifications were found in γS-crystallin, four of which were only seen in the HMW region (i.e., carbamylation of residue I8, deamidations of Q17 and Q93 residues, and one kynurenin at residue W163). Two of these γS-crystallin modifications, N-formylkynurenin at residue W163 and kynurenin at residue W163, were each seen only once in the whole crystallin profile determined from the 69-year-old human lens. Although both CP49 and filensin were identified, CP49 was found in HMW proteins of nuclear region with an oxidation of residue M175. The new PTMs identified in α-, β-, and γ-crystallin, not yet reported in the literature, are highlighted in Table 6.

**Table 6:** Compilation of PTMs found in crystallins and filaments in a 69-year-old human lens.

**Crystallins/filaments**	**Total post-translational modifications**
αA	Acetylation: **M1**
	Ethylation: T13, H79, V89, I146
	Methylation: H79, V89, Q90, I146, Q147, H154, R157
	Oxidation: **M1**, **W9**, H154
αB	Acetylation: M1
	Carbamylation: M1, **K92**, E164
	Deamidation: **N146**
	Ethylation: **H83**, **V93**, **Q108**
	Methylation: **H83**
	Oxidation: **M1**, W9, **W60**, M68**
βA3	Carbamylation: K44, K131
	Deamidation: **Q38**, N40**, Q42**, N103, N133
	Ethylation: W96, M126
	Oxidation: W99, M126
	Sulphone: M126
βA4	Deamidation: N114
	Oxidation: M14
βB1	Deamidation: N68*, N125, Q197, Q236*
	Oxidation: W193
	Phosphorylation: S81
βB2	Acetylation: A2
	Deamidation: Q71, Q105, N114, N116, Q138, Q163, Q183
	Ethylation: **I109**, **K121**, **G161**
	Formylkynurenin***: W85, W151
	Oxidation: W82, **M122**, **H133**, **H135**, W151
γD	Oxidation: M70, M147, W157
γS	Carbamylation: I8**
	Deamidation: Q17**, Q93**, Q171
	Ethylation: I8, K14, K159
	Formylkynurenin***: W163
	Kynurenin***: W163**
	Methylation: H87, K159
	Oxidation: W163
	Phosphorylation: S167
CP49	Oxidation: M175

The asterisk indicates modified species present only in LMW region. The double asterisk indicates modified species present only in HMW region. The triple asterisk indicates Oxidation=Single oxidation; Formylkynurenin=Double oxidation of Trp; Kynurenin=Triple oxidation of Trp. Newly identified PTMs are in bold.

## Discussion

In this study, the experimental approach used a novel combination of LCM, 2D-DIGE, and mass spectrometric analysis. LCM separately recovered proteins from cortical and nuclear regions of a normal human lens, 2D-DIGE determined their comparative distribution, and mass spectrometric analysis determined the identity of crystallins and intermediate filament proteins, and their PTMs in the two regions. The results showed changes in crystallins in fiber cells that occur during their age-related migration from the cortical to nuclear region. Further, the LCM method for isolating cells of the above two regions from lens sections was relatively superior to a similar isolation procedure using sequential solubilization of different regions of a human lens (see Introduction). To minimize crystallin degradation and modification during protein processing, proteins from minimally manipulated tissue sections were fully solubilized in a buffer containing protease inhibitors and denaturating agents. Further, following sensitive 2D-DIGE detection, individual spots were identified based on their amino acid sequences by Q-TRAP LC-MS/MS method (Table 6 and Appendix 1).

Fluorescent dyes (Cy2, Cy3, and Cy5) used in this study, that generally label approximately 1%–2% of Lys residues in proteins, are very useful in determining differential distribution of proteins. CyDye DIGE Fluor minimal dyes contain an N-hydroxysuccinimidyl ester reactive group, which enables labeling of Lys residues within proteins, forming a covalent bond with the epsilon amino group to yield an amide linkage. The recommended concentration of fluor present in a protein labeling reaction ensures that fluor is limiting, and therefore approximately 1%–2% of Lys residues are labeled. As a result, CyDye DIGE Fluor minimal dyes will label only a small proportion of each protein in a sample, hence the expression “minimal labeling” (GE Healthcare Biosciences). Further, minimal labeling of proteins does not affect mass spectrometric data because approximately 98% of protein remains unlabeled. This also means that the technology is not affected by post-translational modifications to Lys residues. For example, if a Lys residue is modified in a tryptic fragment, an adjacent Lys will be alternatively labeled by the dye.

Mass spectrometric analysis of labeled proteins separated by 2D-DIGE identified ~36 spots, which included crystallin fragments, intact crystallins, and crystallin aggregates (referred to as HMW proteins). In this study, only crystallin fragments with M_r_ >10 kDa were analyzed because a filter of 10 kDa was used during protein fractionation (see Methods). Among these, 22 protein spots showed statistically significant differences in abundance levels (p<0.05) between nuclear and cortical regions, as determined by DeCyder analysis.

The major findings of this study were: (A) crystallin fragments (M_r_ <20 kDa) were present in both cortical and nuclear regions, suggesting that the age-related migration of fiber cells from the cortical to nuclear region is accompanied by the truncation of crystallins in the cortical region and continues on in the nuclear region. (B) The HMW aggregates of crystallins (M_r_ >35 kDa) were present in a greater abundance in the nuclear region (i.e., ~3:1 fold difference ratio of nuclear:cortex, Table 3; p<0.05), suggesting that aggregation and/or cross-linking mostly occurred in the nuclear region. (C) The HMW complexes contained a relatively large number of truncated or modified β-crystallin species compared to α- and γ-crystallin, as well as two lens-specific intermediate filaments, CP49 and filensin. (D) The modified α-crystallins were found in low abundance in the nuclear region compared to the cortical region suggesting their chaperone function was possibly compromised due to their truncation and aggregation with other crystallins. This could have potentially resulted in insolubilization of unchaperoned β- and γ-crystallin as seen by their greater abundance in the nuclear region. (E) Several different PTMs (deamidation, oxidation, phosphorylation, methylation, acetylation, ethylation, carbamylation, sulfonation, double and/or triple oxidation, and truncation) of crystallins (αA-, αB-, βA3-, βA4-, βB1-, βB2-, γB-, γC-, γD-, and γS-crystallin) and CP49 and filensin were identified. Some of these PTMs have been identified for the first time in crystallins. Our results show that all lens crystallins undergo post-translational modifications, and these changes would affect their appearance on a 2D-gel. Therefore, crystallins are changing in various directions, and the end result is a difference in overall size of their spot.

To our knowledge, this is the first report in human lens proteomic studies which has used sensitive techniques (LCM and 2D-DIGE) to differentiate the in situ regional distribution of crystallins and intermediate filament proteins in cortical and nuclear regions of the same lens. A previous report examined the distribution of both cortical and nuclear lens proteins in lenses of galgo, beef, cat, deer, rabbit, and chick [48] using a paper and starch gel electrophoretic method [49]. It mainly focused on the functional role of embryonic proteins and changes in their levels during adult life. The application of a combination of LCM, 2D-DIGE, and Q-TRAP LC-MS/MS had a major advantage in that it allowed high throughput analysis and rapid identification of proteins present in lens cortical and nuclear regions. LCM, however, is a time consuming and an expensive technique yielding only a limited amount of protein per 12 μm lens section (i.e., 0.6–2 μg from outer cortical region, 13–15 μg from inner cortical region, and 12–15 μg from the nuclear region, Table 1). To overcome this limitation, cellular proteins from 12 and 48 lens sections were captured by LCM to analyze cortical and nuclear regions, respectively. For LCM, 60–65 h of operation time was consumed, and therefore it became an expensive method for recovering the desired amount of proteins. However, as was evident from the results, the captured cells yielded enough protein for the 2D-DIGE analysis and for the determination of statistically significant differences in abundance in the two lens regions. Additionally, the recovery of sufficient quantities of HMW protein complexes (M_r_ >35 kDa) from the nuclear region (Table 2, Table 4, and Table 5) was more time-consuming and challenging relative to recovery of crystallins with M_r_ <35 kDa from both cortical and nuclear regions. This was because of the existence of HMW proteins at lower levels relative to other crystallin species. Further improvement of the LCM technology, specifically increasing the laser power to cut and catapult a greater number of cells from thicker sections, will make it more cost-effective.

Because PTMs of crystallins are believed to be causative factors for cataract development, we hypothesized that their identification would provide clues regarding the age-related changes in crystallins as cells progressively move from the periphery to the center of the lens. Although several earlier reports have described PTMs in crystallins [12-17,22-25,50-53], it is not clear whether they are localized in cortical or nuclear regions or both.

The presence of truncated and aggregated acidic/basic β-crystallin was relatively higher both in cortical and nuclear regions than α-crystallin, suggesting that β-crystallins were more susceptible to modifications during aging. The β-crystallins showed PTMs in the following decreasing order: deamidation > oxidation > ethylation > carbamylation/ formylkynurenin > phosphorylation/sulphonation/acetylation (Table 6). However, the oxidation of αA- and αB-crystallin occurred more frequently than other PTMs. αA-Crystallin also exhibited maximum methylation compared to other crystallins, which has been reported for the first time here. Another interesting finding was that only truncated forms of βB2-crystallin were present in both nuclear and cortical regions, suggesting that modified βB2-crystallin is more susceptible to degradation than other β-crystallins. Further, mass spectrometric analysis showed that truncated crystallins with M_r_ <20 kDa were recovered from both cortical and nuclear regions (Table 2, spot numbers 1 to 11B), and these spots were considered as crystallin fragments. DeCyder analysis also showed that certain crystallin fragments were more abundant in the cortical region compared to the nuclear region, suggesting a greater truncation of certain crystallins in the cortical region relative to the nuclear region.

NH_2_-terminal acetylation, a widespread PTM in eukaryotes and viruses compared to prokaryotes [54], is believed to protect proteins against proteolytic degradation by aminopeptidases. More than two decades ago, Driessen et al. [55] reported the presence of NH_2_-terminally acetylated α/β-crystallins in calf lens, while others reported it in human [25] and chicken lenses [56]. However, its lenticular localization was still unknown. Our results showed for the first time that NH_2_-terminally acetylated αA- and αB-crystallins are present in both cortical and nuclear regions of human lens. NH_2_-terminal acetylation (e.g., M1 acetylation) was reported earlier in human αA- and αB-crystallins in water soluble and insoluble fractions using whole lens extracts [25]. Lin et al. [57] have reported in vivo acetylation at Lys 70 in human αA-crystallin and proposed that it may affect its chaperone function. Similarly, acetylation at Ala 2 in βB2-crystallin, as shown in the present study, may alter surface charge, protein conformation and intermolecular interaction. The functional significance of NH_2_-terminal acetylation is presently unknown but since NH_2_-termini of αA- [32] and αB-crystallin (Asomugha and Srivastava, unpublished results) play a major role in maintaining structural integrity and solubility, acetylation of NH_2_-termini may therefore alter oligomeric structure and affect chaperone function.

Deamidation, which induces a net “charge-change” by conversion of a neutral amide group to an acidic group in asparaginyl and glutaminyl residues of crystallins, alters structural and functional properties. Although the deamidated α-crystallins were absent in the HMW complex, they were present as crystallin fragments (M_r_ <20 kDa). This study identified a significant number of deamidated β-crystallin species in the HMW complex of the nuclear region, suggesting that these deamidated crystallin species aggregate with aging. Also, deamidation was identified as one of the major and most abundantly occurring PTMs in β-crystallins, with greater deamidation of Gln residues than Asn residues as reported earlier by Hains et al. [23]. βA3-crystallin with deamidation of Q38, N40, Q42, N103, and N133 residues were identified, but only species with deamidations at Q38, N40, and Q42 were present in the nuclear region. Furthermore, deamidations of N40, N103, and N133 sites in βA3-crystallin have been reported for the first time. We also identified novel and previously unreported deamidation sites in other β-crystallins (βA4 – N114; βB1 – N68, N125, Q197, Q236; βB2 – Q71, Q105, N114, N116, Q138, Q163, Q183). While deamidated βA4- and βB1-crystallin were present in both cortical and nuclear regions, deamidated βB2-crystallin was present only in the cortical region. Although the effects of different deamidations reported for the first time is presently unclear, previous reports by Lampi et al. [22], along with ourselves [20,21], have demonstrated deleterious effects of specific deamidations of Asn and Gln residues on structural/functional properties of crystallins. Understanding the effects of the above-mentioned deamidations would provide insight into the mechanism of crystallin destabilization and their age-related aggregation.

Another major PTM identified in the present study was oxidation of M, H, and W residues. Again, β-crystallin showed relatively greater levels of oxidatively modified species than α-crystallin. Oxidation of M68 in αB-crystallin was observed, as reported previously in both WS- and WI-protein fractions of aging and cataractous human lenses [26,27]. Additional oxidation sites observed were: αA – M1, W9, H154; αB – M1, W9, W60, M68; βA3 – W99, M126; βA4 – M14; βB1 – W193; βB2 – W85, M122, H133, H135, W151; γS – W163. Because methionine oxidation might occur during sample handling, some of the observed Met oxidations could be artifacts. These oxidized crystallin species were present in both the crystallin fragment fraction (M_r_ <20 kDa) and the aggregated crystallin fraction (M_r_ >35 kDa). Although it was unclear if truncation and aggregation of crystallins were results of their oxidation, their presence in the above two fractions was a hallmark of the aging process. Oxidized γD-crystallin (oxidized at M70, M147, and W157 residues) was present in the HMW complex and was localized only in the nuclear region, suggesting that the oxidation of γD-crystallin resulted in its aggregation in the nuclear region. A previous report [58] showed that γ-crystallins migrated as aggregated species if not boiled with SDS-gel loading buffer. That study used in vitro synthesized γ-crystallins, unlike this study, but whether this was a factor in the present study was not determined. Our findings of doubly- and triply-oxidized W (formylkynurenin and kynurenin) observed in βB2-crystallin residues W85 and W151 [59] and W163 residue of γS-crystallin further suggest that oxidation of W might lead to aggregation of β- and γ-crystallins. Present literature suggests that UVA-exposure induces production of reactive oxygen species, which oxidizes M, H and W residues of crystallins and would result in their structural alterations and aggregation. For example, triply-oxidized W residues in these crystallins may have reacted with either the ground state molecular oxygen to form ^1^O_2_ or with amino acids to produce free radicals, which might have resulted in the presence of modified species in the HMW complex.

Phosphorylation, a known developmental regulatory element in a variety of proteins including lens crystallins, was found to be associated with aging [60]. An interesting finding of the present study was that only S81 and S167 residues of βB1- and γS-crystallins, respectively, were identified as phosphorylated species in the HMW complex of the nuclear region. Several phosphorylated sites in αB-crystallin have been previously reported and some of these were present in a cataractous lens [30]. At present, the effects of phosphorylation on aggregation properties of βB1- and γS-crystallins are not known.

The present study demonstrated carbamylation of M, E, K, and I residues in human αB-, βA3- and γS-crystallins. All these carbamylation sites in the crystallins have been identified for the first time, with the exception of the K92 residue in αB-crystallin identified as a carbamylation site in calf lens [19].

In summary, we have identified several new PTM sites in crystallins and intermediate filament proteins in the cortical and/or nuclear regions. We also showed that truncation of crystallins began in the cortex and continues in the nuclear region, whereas crystallin aggregation is mostly localized in the nuclear region. The study showed that α-, β-, and γ-crystallins exhibited several sites of deamidation (N and Q), oxidation (M, H, W), methylation (H, V, Q, I, R and K) and carbamylation (M, E, K and I), and these modified species were present in both cortical and nuclear regions of an aging lens. Together these PTMs might disrupt the net charges and conformations of the crystallins, and lead to protein degradation, aggregation, or both. Because deamidation [20,21,32] and oxidation [4,14] have been shown to affect chaperone activity of α-crystallins, the apparent loss of α-crystallin chaperone function with aging might leave β- and γ-crystallins more susceptible to degradation and aggregation. Additionally, we have successfully performed the proteomic analysis of a human lens by tissue microdissection, 2D-DIGE separation and mass spectrometric analysis. These methods would be helpful in the future in analyzing cataractous lenses to distinguish age-related events from those occurring during cataract development. Because of the use of only two lenses, it remains to be seen whether these relative changes in crystallins in cortical versus nuclear regions are universal.

## Appendix

Crystallin species, sequences, and modifications from Coomassie-stained 2D-DIGE gel of a 69-year-old human lens identified by ESI-QTRAP LC-MS/MS. To access the data, click or select the words “Appendix 1.” This will initiate the download of a compressed (pdf) archive that contains the file.
